# GqqA, a novel protein in *Komagataeibacter europaeus* involved in bacterial quorum quenching and cellulose formation

**DOI:** 10.1186/s12934-016-0482-y

**Published:** 2016-05-24

**Authors:** Maria José Valera, Albert Mas, Wolfgang R. Streit, Estibaliz Mateo

**Affiliations:** Biotecnología Enológica. Dept. Bioquímica i Biotecnologia, Facultat d‘Enologia, Universitat Rovira i Virgili, C/Marcel.lí Domingo s/n., 43007 Tarragona, Spain; Abteilung für Mikrobiologie und Biotechnologie, Biozentrum Klein Flottbek, Universität Hamburg, Ohnhorststr. 18, 22609 Hamburg, Germany; Departamento de Inmunología, Microbiología y Parasitología, Facultad de Medicina y Odontología, Universidad del País Vasco/Euskal Herriko Unibertsitatea UPV/EHU, Barrio Sarriena s/n., 48940 Leioa, Spain

**Keywords:** Homoserine lactone, Acetic acid bacteria, Vinegar, Quorum quenching, Genomic fosmid library

## Abstract

**Background:**

We report on the functional screening and identification of an active quorum quenching (QQ) gene in the *Komagataeibacter europaeus* strain CECT 8546, which is a member of the acetic acid bacteria (AAB).

**Results:**

Using a previously published screening protocol (Schipper et al., in Appl Environ Microbiol 75:224–233, 2009. doi: 10.1128/AEM.01389-08) for QQ genes, we identified a single gene, designated *gqqA*, that interfered strongly with bacterial quorum sensing (QS) in various reporter strains. It encodes for a 281-amino acid protein with a molecular mass of 30 kDa. Although the GqqA protein is similar to predicted prephenate dehydratases, it does not complement *Escherichia coli* mutants of the *pheA* gene, thus indicating a potentially different function. Recombinant GqqA protein attenuated QS-dependent pyocyanin production and swarming motility in the *Pseudomonas aeruginosa* strain PAO1. Moreover, GqqA quenched the QS response of the *Agrobacterium tumefaciens* NTL4 and the *Chromobacterium violaceum* CV026 reporter strains. Interestingly, the addition of recombinant GqqA protein to growing cultures of the *Komagataeibacter europaeus* strain CECT 8546 altered the cellulose production phenotype of CECT 8546 and other AAB strains. In the presence of GqqA protein, cells were planktonic, and no visible cellulose biofilms formed. The addition of low levels of *N*-acylhomoserine lactones maintained the biofilm formation phenotype.

**Conclusions:**

Our data provide evidence for an interconnection between QS and AAB cellulose biofilm formation as well as QQ activity of the GqqA protein.

**Electronic supplementary material:**

The online version of this article (doi:10.1186/s12934-016-0482-y) contains supplementary material, which is available to authorized users.

## Background

Quorum-sensing (QS) is a cell density-dependent system that involves the coordinated expression of genes to regulate diverse physiological functions in microorganisms such as motility, production of extracellular proteins, biofilm formation, pathogenicity, and others throughout the majority of cells within an isogenic population [1, 2]. This cell–cell communication is mediated by autoinducers such as* N*-acylhomoserine lactones (*N*-AHLs), which are the best-characterized quorum signals produced, by many Gram-negative bacteria, and their general mechanism of synthesis is well understood [3]. In recent years, more than 20 molecules from QS and non-QS microbes have been reported as quorum quenchers for their capacity to interfere with these autoinducers [4–7]. They can act as antagonists of the native autoinducer or as enzymes that catalyze the degradation of the autoinducer molecule and thereby inhibit QS signaling [4, 7]. However, the physiological activity of most of these quorum-quenching (QQ) molecules is not clear [8].

Acetic Acid Bacteria (AAB) are a group of Gram-negative aerobic bacteria within the *Acetobacteraceae* family. They are involved in the partial oxidation of carbohydrates and alcohols and the release of organic acids as end products into the media [9]. AAB are largely known for their ability to produce acetic acid on ethanol-containing substrates, resulting in vinegar. In the production of vinegar by the traditional method, AAB tend to be placed on the air–liquid interface, developing a cellulose biofilm, to be in direct contact with oxygen [10, 11] and likely also to survive under stress conditions such as high ethanol or acetic acid concentrations [12]. Recently, the presence of a QS* N*-AHL-dependent system termed GinI/GinR, which is homologous to LuxI/LuxR described in *Vibrio fischeri*, has been reported for *Komagataeibacter intermedius* (formerly *Gluconacetobacter intermedius*) [13]. In this species, three different AHL molecules with different acyl chains have been described:* N*-decanoyl-l-homoserine lactone (*N*-C_10_-HSL), *N*-dodecanoyl-l-homoserine lactone (*N*-C_12_-HSL) and C_12_-HSL, which has a single unsaturated carbon bond [13]. Via these AHLs, the GinI/GinR QS system is involved in the repression of acetic acid and gluconic acid production as well as antifoam activity [14, 15]. Although there is some knowledge of QS in the AABs, nothing is known regarding the QQ mechanism within this group of bacteria.

Within this framework, we were interested to identify possible QQ genes in the strain *Komagataeibacter europaeus* CECT 8546 (formerly *Gluconacetobacter europaeus*) [16]. CECT 8546 is a cellulose overproducer and biofilm-forming strain that was isolated from vinegar elaborated by the traditional method. In the present study, we provide evidence that this strain encodes at least a single QQ gene, which we designated GqqA. The GqqA protein is similar to predicted prephenate dehydratases and it interferes with biofilm formation in CECT 8546 and other closely related strains.

## Results

### Detection of QS interfering clones and genetic analysis

**Fig. 1:**
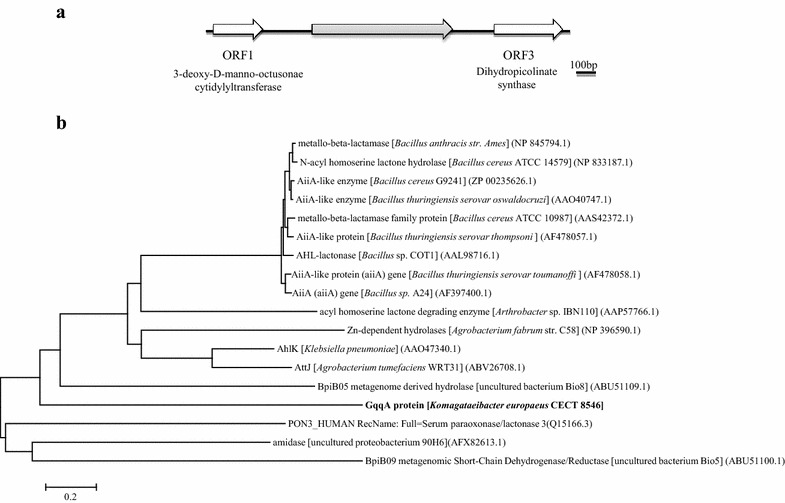
**a** Physical map of the predicted ORFs in the insert (1.8 kb) obtained after subcloning, which contained the selected positive QQ clone. The position and the direction of transcription are indicated for ORFs. **b** Phylogenetic analysis of the GqqA protein and 17 proteins with described QQ activity. Amino acid sequences were obtained from NCBI GenBank, and accession numbers are in *brackets*. The dendrogram was constructed after ClustalW alignment using the neighbor-joining and Kimura two-parameter methods subjected to 1000 bootstrap trials and conducted with MEGA software version 4 [47, 48]

**Fig. 2:**
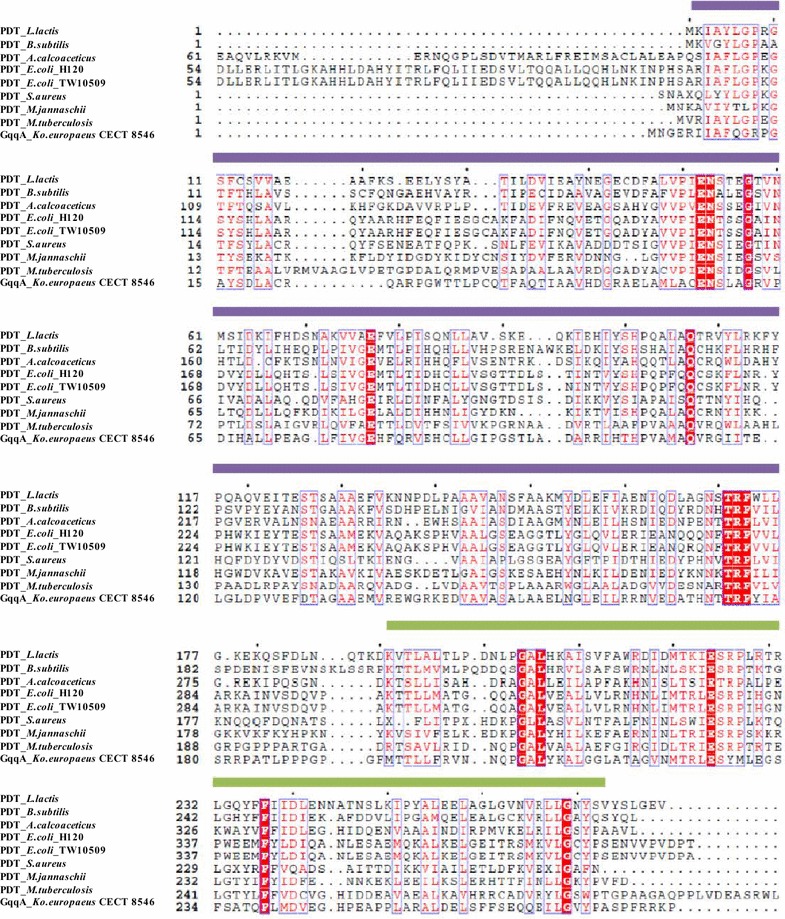
Amino acid sequence alignment of the GqqA protein and eight PDT proteins characterized enzymatically (GenBank accession number in *brackets*) from *Lactococcus lactis* (CAA55182.1), *Bacillus subtilis* (AAA22507.1), *Acinetobacter calcoaceticus* (AAA22507.1), *Escherichia coli* H120 (EGB42307.1), *E. coli* TW10509 (EGB73575.1), *Staphylococcus aureus* Mu50 (2QMW_A), *Methanocaldococcus jannaschii* DSM2661 (Q58054.1), and *Mycobacterium tuberculosis* H37Ra (ABQ75667.1). Strictly conserved residues are highlighted with *red boxes*; *red characters* indicate similarity in a group and *blue frames* indicate similarity across groups. Regions homologous to the PDT family are marked with a *purple box*, and the *green box* covers the ACT domain. The sequence alignments were assembled using ClustalW and visualized using ESPript software (http://espript.ibcp.fr/ESPript/cgi-bin/ESPript.cgi)

To identify possible QQ genes in CECT 8546, we initially constructed a fosmid library using established protocols. The library encompassed 1824 fosmid clones, and clones had average insert of 35 kb (Additional file 1: Figure S1A). The clones were tested using the reporter strain NTL4 of *Agrobacterium tumefaciens* carrying a *traI*–*lacZ* reporter gene (AT soft agar screening) for QQ activities. A total of 13 fosmid clones consistently gave a positive result for QS inhibition in AT soft agar medium, and seven of them were digested and subcloned. The obtained subclones were tested again with the strain NTL4 in AT soft agar medium. From this initial screening, 16 positive clones were analyzed using the reporter strain PAO1 of *Pseudomonas aeruginosa* for pyocyanin production and the transformed strain DH5α of *Escherichia coli* for motility tests. All of the clones were sequenced and compared with the NCBI database (data not shown). Two clones were selected because they presented the same sequence as well as a strong and reproducible QS inhibiting phenotype in the assays performed. The sequence insert in these two clones, with a size of 1.8 kb, was analyzed. Three ORFs were detected: ORF1 encoded for a predicted 3-deoxy-d-manno-octulosonate cytidylyltransferase, ORF2 encoded for possible prephenate dehydratase and ORF3 encoded for predicted dihydrodipicolinate synthase. The DNA sequence of ORF2, which was 846 bp, was designated *gqqA* and corresponded to 281 amino acids (Fig. 1a). The highest similarity was found on the amino acid level of the strain LMG 18494 of *Komagataeibacter europaeus* (GenBank accession number: WP_010507907.1) with a predicted prephenate dehydratase (PDT) protein. The similarity observed was 100 % at the amino acid level. Interestingly, no conserved domains known to be involved in lactonases or any other described AHL-degrading molecule were identified in the amino acid sequence of the GqqA protein (Fig. 1b). However, this protein was found to have a periplasmic binding protein domain type 2 superfamily at its N-terminus and an ACT superfamily (ACT-CM-PDT) domain at its C-terminus. The ACT domains usually are regulatory domains that bind an allosteric effector. They are related with the binding of small molecules such as amino acids. The periplasmic binding domain is the catalytic domain involved in signal perception such as nutrient uptake or chemotaxis [17, 18]. An alignment of the sequence of GqqA with PDT amino acid sequences of other microorganisms indicated that GqqA carried several conserved residues that are present in the homologous regions of PDT family proteins. These residues corresponded to amino acid residues 6 to 183 and an ACT domain between residues 195 and 273 present in all of the PDT sequences (Fig. 2).

In general, PDTs are involved in the metabolic pathways of the aromatic amino acids. They convert prephenate to phenylpyruvate in the biosynthesis of l-phenylalanine. To confirm this identity and corroborate the proposed activity for this enzyme, two defective *E. coli* strains (JW2580-1 and KA197) for the gene *pheA* obtained from the *E. coli* Genetic Stock Center were used for the complementation assay. Both *E. coli* mutants were transformed with the plasmid pET21a::*gqqA*. They were not able to grow in M9 minimal medium, but supplementing this medium with phenylalanine allowed their growth. This result indicated that the activity of GqqA did not coincident with the predicted PDT enzyme function.

### Heterologous expression of GqqA protein and activity tests using the reporter strains of *Agrobacterium tumefaciens* NTL4, *Pseudomonas aeruginosa* PA01 and *Chromobacterium violaceum* CV026

To further characterize the function of the GqqA protein, we heterologously expressed the *gqqA* gene in *E. coli* BL21 (DE3). For this, a DNA fragment of 846 bp carrying the *gqqA* gene was amplified by PCR and inserted into the expression vector pET-21a. The correctness of the construct was verified by sequencing. The protein was induced for recombinant expression with IPTG in the strain BL21 (DE3) of *E. coli*, purified from the soluble fraction, and finally, analyzed by SDS-PAGE; the results indicated that the protein was homogenous, with only minor contamination by other proteins (Additional file 1: Figure S1B). The purified protein presented a molecular weight of approximately 30 kDa, which was in accordance with the calculated molecular weight of 30.52 kDa.

**Fig. 3:**
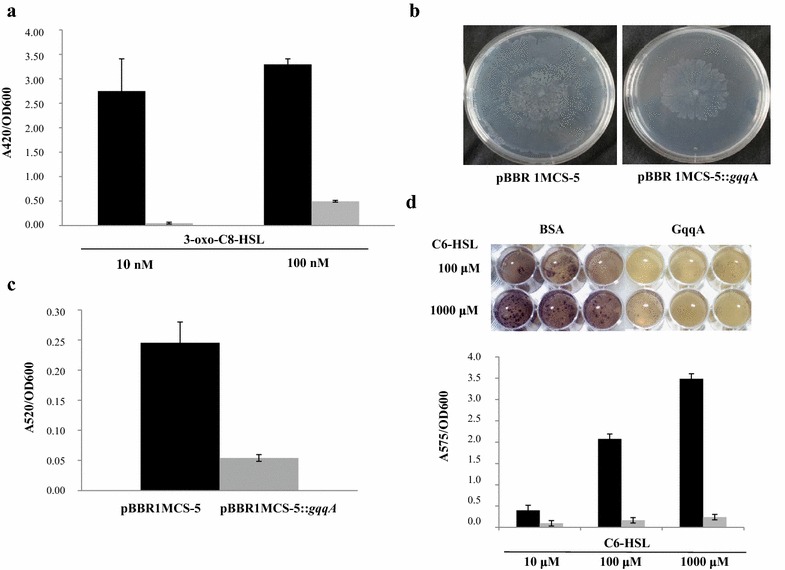
**a** β-Galactosidase activity of the strain NTL4 of *A. tumefaciens* incubated with different *N*-AHLs and using the ONPG substrate; *values* indicate relative absorbance from measurements at 420 nm relative to the cellular growth at 600 nm. *Gray bars* represent samples incubated with the GqqA protein and *black bars* with BSA as a control. *Error bars* represent standard deviations. Data are mean values of at least three measurements. **b** Motility tests performed on swarming agar with the strain PAO1 of *P. aeruginosa* transformed with pBBR1MCS-5::*gqqA* and the re-circularized vector pBBR1MCS-5. **c** Pyocyanin production of the strain PAO1 transformed with pBBR1MCS-5::*gqqA* and pBBR1MCS-5 empty vector, values of relative absorbance from measurements at 520 nm relative to the cellular growth at 600 nm. *Error bars* represent standard deviations. Data are mean values of at least three measurements. **d** Difference in violacein production by the strain Cv026 of *C. violaceum* using different concentrations of C6-HSL in the presence of the GqqA protein and BSA (15 µg/ml), shown in triplicate. Relative quantification of the violacein produced under different conditions measured by the absorbance at 575 nm relative to the cellular growth at 600 nm. *Gray bars* represent samples incubated with the GqqA protein and *black bars* with BSA as a control. *Error bars* represent standard deviation. Data are mean values of at least three measurements

The activity of the GqqA protein was assayed using the *A. tumefaciens* NTL4 reporter strain. In the presence of GqqA, a decrease in the absorbance was detected with the ONPG test, which corresponds with a decrease in the added *N*-AHLs. Two different concentrations of 3-oxo-C8-HSL molecules were incubated for 2 h with the GqqA protein, and in both cases the levels of detected *N*-AHLs were significantly reduced in comparison to the controls (Fig. 3a). The reduction in β**-**galactosidase activity ranged between 85 and 98 %.

Consistent with the above observations, the expression of the *gqqA* gene in the *P. aeruginosa* strain PAO1 affected motility and pyocyanin production. For this purpose, the strain PAO1 was transformed with the construct pBBR1MCS-5::*gqqA*, and a control plasmid consisting of the empty broad host vector pBBR1MCS-5 was used. Sequencing verified the correctness of the *gqqA* insert in the vector. The presence of the *gqqA* gene had a strong effect upon the motility inhibition tested on swarming agar in comparison to the control (Fig. 3b). Pyocyanin pigment production was also reduced in strain PAO1 transformed with pBBR1MCS-5::*gqqA*. A reduction of 88 % (relative absorbance measured at 520 nm) was detected for pyocyanin production with respect to the control (Fig. 3c).

These results were supported by data obtained with the *C. violaceum* CV026 reporter strain. These tests implied that GqqA protein was highly active in reducing the QS-dependent production of violacein compared to the control (Fig. 3d). Using 100 μM of added C6-HSL, the QQ activity was clearly detectable, even visually. Nevertheless, a reduction in violacein production of 75.6 % using 10 μM C6-HSL was observed, a reduction of 91.9 % when 100 μM C6-HSL was added, and 93.1 % when the highest amount of C6-HSL, which corresponded to 1 mM, was tested.

Altogether, these tests indicated that the GqqA protein degraded or modified the AHL molecule in such a way that it was not detected by any of the reporter strains used.

### GqqA impact on the formation of cellulose aggregates in the strain CECT 8546 and other AAB strains

**Fig. 4:**
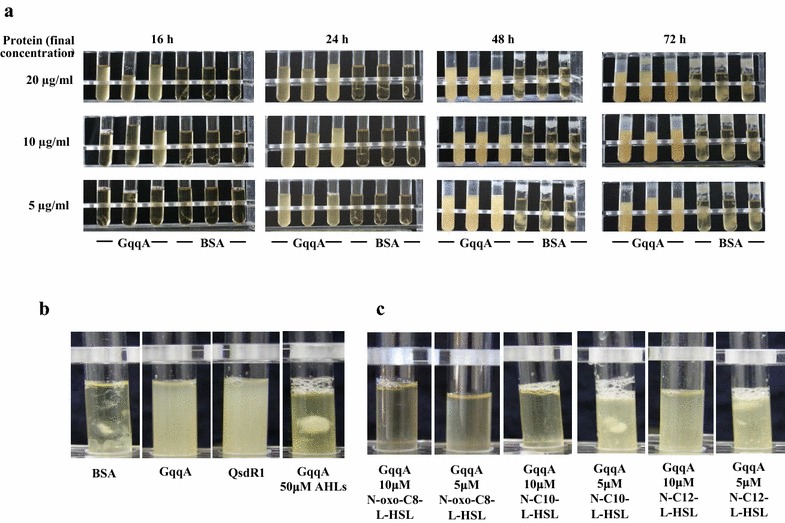
Growth and cellulose production phenotypes of the strain CECT 8546 of *Komagataeibacter europaeus.* **a** with three different concentrations (5, 10, and 20 μg/ml) of the GqqA protein and BSA as a control after 16, 24, 48 and 72 h. **b** BSA as control, GqqA protein (20 μg/ml), QsdR1 protein (20 μg/ml), and GqqA protein (20 μg/ml) supplemented with the AHL molecules *N*-oxo-C8-l-HSL, *N*-C10-l-HSL and *N*-C12-l-HSL (50 μM of each) after 24 h. **c** GqqA protein (20 μg/ml) supplemented with individual AHL molecules (*N*-oxo-C8-l-HSL, *N*-C10-l-HSL and *N*-C12-l-HSL) at a final concentration of either 5 or 10 μM. All of these experiments were performed in triplicate

The strain CECT 8546 usually forms strong cellulose aggregates, which are visible after 16 h of growth in GY medium. To test if GqqA affects aggregate formation, this recombinant protein was added at three different concentrations to the growth medium. The growth behavior of GqqA-treated cells was clearly different from the BSA controls (Fig. 4a). The turbidity of the culture increased through time in the presence of the GqqA protein, while the control cultures did not exhibit any altered behavior, forming cellulose aggregates. The addition of 20 μg/ml of GqqA protein caused the strongest phenotype and the addition of only 5 μg/ml of protein still resulted in a clear phenotype but with a slight time delay (Fig. 4a). This phenotype was also observed when the QsdR1 protein from *Rhizobium* sp. strain NGR234, which was previously reported to act as a lactonase on AHLs [19], was added (Fig. 4b). In addition to BSA, protein extract from *E. coli* was also tested as controls and no difference was detected (data not shown). Moreover, as an additional control, we added 3-oxo-C8-HSL, *N*-C10-l-HSL and *N*-C12-l-HSL in addition to the GqqA protein to the CECT 8546 strain cultures. Interestingly, strain CECT 8546 grew and formed cellulose aggregates with the addition of *N*-AHLs (Fig. 4b). The best results were obtained with 3-oxo-C8-HSL, although the addition of either the *N*-C10-l-HSL or the *N*-C12-l-HSL molecules also produced this phenotype (Fig. 4c).

**Fig. 5:**
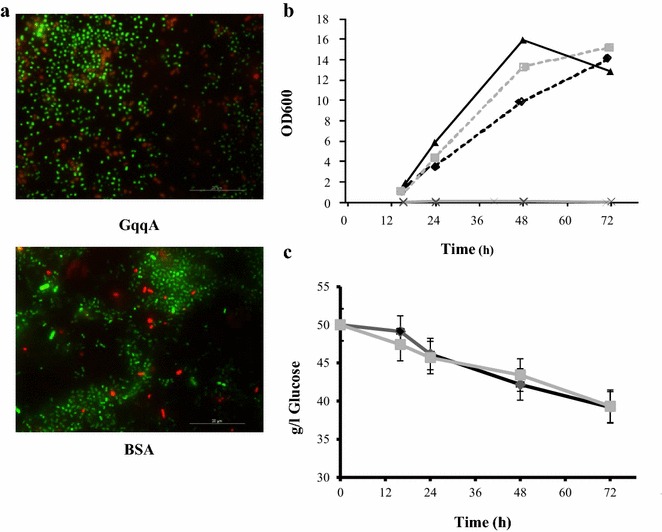
**a** Cell disposition of cultures of the strain CECT 8546 of *Komagataeibacter europaeus* after 16 h with the GqqA protein or BSA visualized by epifluorescence microscopy with the Live/Dead BacLight Kit. **b** Turbidity measurements at 600 nm during the growth of the strain CECT 8546 of *Komagataeibacter europaeus* at different concentrations of the GqqA protein: 20 μg/ml (*black solid line*), 10 μg/ml (*gray dotted line*), 5 μg/ml (*black dotted line*), and 5 μg/ml of BSA as a control (*gray solid line*). **c**) Glucose decrease in the medium GY during the growth of the strain CECT 8546 of *Komagataeibacter europaeus* with either 20 μg/ml of GqqA protein (*gray line*) or BSA (*black line*). *Error bars* represent standard deviations. Values are the mean of three measurements

It is noteworthy that further analysis of cells treated with the GqqA protein and the controls, which contained BSA or protein extract from *E. coli* instead of GqqA protein, largely confirmed the above observations. Under the microscope, cells treated with GqqA were mostly planktonic, while control cultures were associated in biofilms (Fig. 5a). Furthermore, confirming the turbidity measurements (Fig. 5b), cell counts indicated that in the presence of the GqqA protein, the total cell population as determined by microscopy counts ranged from 10^8^ cells/ml (24 h) to 5·10^9^ cells/ml after 72 h of growth, and the colony forming units ranged from 8·10^7^ cfu/ml (24 h) to 3.5·10^9^ cfu/ml (72 h). For the control culture, the microscopy counts were more difficult to obtain due to the presence of aggregates. However, cell counts yielded cell populations of approximately 2·10^5^ cells/ml (24 h) to 10^6^ cells/ml after 72 h of growth and within the biofilms. The colony forming unit counts ranged from 2.3·10^5^ cfu/ml (24 h) to 7.9·10^5^ cfu/ml (72 h).

Furthermore, we measured the amount of d-glucose in the medium to exclude the possibility that the addition of the GqqA protein would modify glucose levels. No significant differences were observed between those cultures that were supplemented with GqqA protein and the controls (Fig. 5c).

Altogether, these data imply that the GqqA protein exerts a strong impact on cellulose-biofilm formation by the strain CECT 8546 and that this effect is attributable to alterations in the produced AHL molecules and not to the fact that glucose levels were altered.

**Fig. 6:**
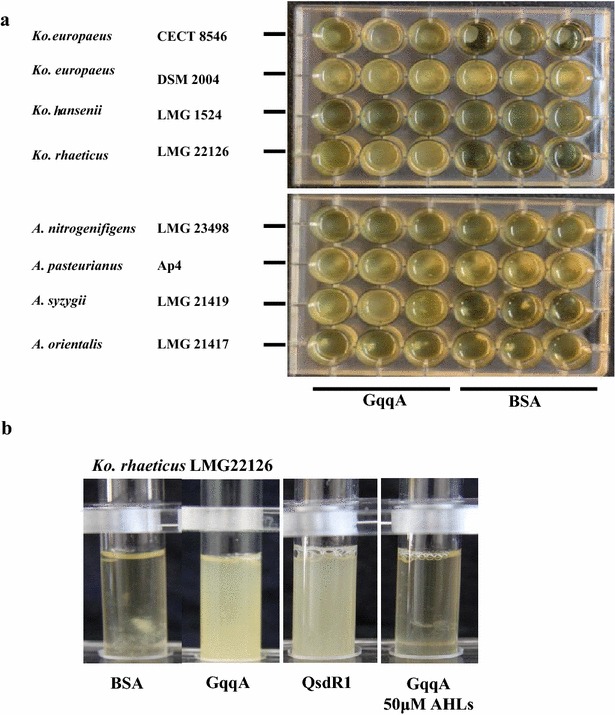
**a** Growth and cellulose production phenotypes of eight AAB strains with the GqqA protein and BSA as a control (50 μg/ml in both cases). **b** Growth and cellulose production phenotypes of the strain *Komagataeibacter rhaeticus* LMG22126 incubated with BSA as a control, GqqA protein (20 μg/ml), QsdR1 protein (20 μg/ml), and GqqA supplemented with the AHL molecules *N*-oxo-C8-l-HSL, *N*-C10-l-HSL and *N*-C12-l-HSL (50 μM for each)

Moreover, seven additional strains of AABs, selected as cellulose producers, were analyzed with respect to their growth and biofilm phenotypes in the presence of the GqqA protein (Fig. 6a). In the case of the strains *Komagataeibacter europaeus* DSM 2004, *Komagataeibacter hansenii* LMG 1524, *Acetobacter nitrogenifigens* LMG 23498 and *Acetobacter orientalis* LMG 21417, no visible changes in their growth phenotypes were detected with respect to a BSA control. The strain Ap4 of *Acetobacter pasteurianus* produced a similar turbidity, but cellulose aggregation was more clearly visible in the control. The growth of strains *Acetobacter syzigii* LMG 21419 and *Komagataeibacter rhaeticus* LMG 22126 was more turbid in the presence of the GqqA protein. Furthermore, the addition of the QsdR1 protein resulted in the same growth phenotype for the strain *Komagataeibacter rhaeticus* LMG 22126 as that produced in the presence of the GqqA protein (Fig. 6b). Moreover, the addition of 3-oxo-C8-HSL, *N*-C10-l-HSL and *N*-C12-l-HSL to cultures containing the GqqA protein resulted in a cellulose-biofilm phenotype as was observed for the strain CECT 8546.

These results further suggest that, in addition to the effects observed in the strain CECT 8546, the GqqA protein also affects cellulose production in other AAB strains. Therefore, this may indicate that the GqqA protein interferes in AAB cellulose formation.

## Discussion

AAB are primarily known to be involved in vinegar production, in which they develop a biofilm at the air–liquid interface, generally when vinegar production is carried out with the traditional method [9]. In the last few years, an *N*-AHL-dependent QS mechanism designated as the GinI/GinR system in *Komagataeibacter intermedius* has been reported to be responsible for the repression of acetic acid and gluconic acid production, antifoam activity, and growth rate acceleration in the exponential growth phase [13–15]. This system is regulated by long chain *N*-AHL molecules such as *N*-C10-l-HSL and N-C12-l-HSL [13].

Despite current knowledge of the QS systems of AAB bacteria, no studies have yet been published examining QQ activities. Therefore, in this work, the first screening for QQ activity was carried out from the genome of *Komagataeibacter europaeus* CECT 8546, a cellulose-overproducing AAB strain [16]. A protein named GqqA was identified within a fosmid library of this strain, and its potential QQ role was characterized. Thereby, tests using the reporter strains *A. tumefaciens* NTL4 and *C. violaceum* Cv026 confirmed the QQ activity of the GqqA protein. Furthermore, we provided evidence that the GqqA protein affected QS-dependent processes in the *P. aeruginosa* strain PAO1, such as motility and pyocyanin production. Although the molecular mechanism by which GqqA acts on the AHL molecules is not yet known, these results suggest a modification of the QS mechanisms from the reporter strains.

Additional assays were performed to test the possible effects of the GqqA protein on the growth and physiology of the strain CECT 8546 and other AAB cellulose-producing strains. Generally, the cells of the CECT 8546 strain tended to aggregate in a cellulose biofilm, but interestingly, in the presence of the GqqA protein, no cellulose aggregates were formed, and the turbidity of the medium increased. This observation was not only obtained for strain CECT 8546 of *Komagataeibacter europaeus* but also for other cellulose-producing strains belonging to the *Acetobacter* and *Komagataeibacter* genera. These results imply that the GqqA protein exerts an effect on the cellulose production of AAB strains.

Because cellulose production during vinegar production is industrially undesirable [20, 21], the finding that the GqqA protein interferes with cellulose production, at least in some AAB strains, is of biotechnological relevance. Moreover, these results could also contribute to further knowledge of the synthesis mechanism for this polymer in AAB.

Conversely, as far as we know, there is no evidence of QS control for cellulose biofilm formation in AAB. However, it is well known that there is an inverse relationship between gluconic acid production and cellulose formed in this bacterial group and how these pathways are connected with sugar metabolism [22]. Moreover, it has been reported that gluconic acid biosynthesis is controlled by QS systems in *Komagataeibacter intermedius* [13]. Altogether, these data indicate a role for QS in cellulose formation in AAB.

There are three main types of microbial enzymes whose activity has been demonstrated in *N*-AHL signaling interference: oxidoreductases, acylases, and lactonases [7, 8]. The best characterized group of enzymes able to cleave the *N*-AHL molecules are lactonases, which can hydrolyze the lactone ring in a reversible way [7]. The phylogenetic analysis performed with the amino acid sequences of the GqqA protein and those of known QQ proteins grouped the GqqA protein in a separate cluster from the other QQ proteins. The predicted amino acid sequence of the GqqA protein as well as the DNA sequence of the ORF presented the highest homologies with predicted PDTs from AAB. Curiously, the GqqA protein was only faintly similar to PDT enzymes that had been functionally verified; a homology of 31 % with the PDT sequence of the strain *E. coli* H120 (EGB42307.1) was observed. Moreover, the complementation assays performed with two *E. coli* mutants of the *pheA* gene revealed that the *gqq*A gene could not restore these auxotrophic strains. These observations suggest a different function for the GqqA protein in the CECT 8454 strain and in other AAB.

## Conclusions

The data provided within this work imply a noticeable effect of the GqqA protein on cellulose biofilm production for the strain CECT 8546 of *Komagataeibacter europaeus* and other AAB strains. This is a novel finding, and no report has been published in which a protein with light homology to other described AHL-degrading molecules and with high similarity to PDT enzymes presents QQ activity. Further work is necessary to elucidate the mechanisms and the regulatory circuits of this potential QQ protein.

## Methods

### Bacterial strains, plasmids and culture conditions

In the present study, different bacterial strains and constructs were used (Table 1) [23–35]. The strains DH5α, EPI300™ and BL21 (DE3) of *Escherichia coli* as well as the strain PAO1 of *Pseudomonas aeruginosa* were grown in LB medium (1 % tryptone; 0.5 % NaCl; 0.5 % yeast extract) at 37 °C. Antibiotics were added in the medium depending on the vector used: chloramphenicol (final concentration of 12.5 mg/ml) for clones containing the fosmid vector CopyControl pCC1FOS™ Vector, ampicillin (final concentration 100 mg/ml) for clones containing the vector pBlueScript II SK (+) and the vector pET-21a, and gentamycin (final concentration 50 mg/ml) for clones containing the broad host range vector pBBR1MCS-5.

**Table 1:** Bacterial strains, constructs and vectors used in this study

Bacterial strain	Description	Reference or source
*Escherichia coli* EPI300™	F^−^ *mcrA Δ(mrr*-*hsdRMS*-*mcrBC) Φ80dlacZΔM15 ΔlacX74 recA1 endA1 araD139 Δ(ara, leu)7697 galU galK λ* ^−^ *rpsL (StrR) nupG trfA dhfr*	Epicentre biotechnologies, Madison, WI
*Escherichia coli* DH5α	F^−^ Φ80*lac*ZΔM15 Δ(*lac*ZYA-*arg*F) U169 *rec*A1 *end*A1 *hsd*R17 (rK^–^, mK^+^) *pho*A *sup*E44 λ– *thi*-1 *gyr*A96 *rel*A1	Invitrogen, Karlsruhe, Germany [23]
*Escherichia coli* BL21(DE3)	F^−^ ompT hsdS_B_ (r_B_^−^ m_B_^−^) gal dcm (DE3)	Novagen, Darmstadt, Germany
*Pseudomonas aeruginosa* PAO1	Wild type strain; Amp^r^	Holloway et al. [24]
*Agrobacterium tumefaciens NTL4 (pCF218)(pCF372)*	Reporter strain for AHL detection; traI::lacZ Tet^r^ Sp^r^	Fuqua et al., Luo et al. [25–27]
*Chromobacterium violaceum CV026*	Reporter strain for autoinducer I; mini-Tn5 in *cvi*I	McClean [28]
*Escherichia coli* KA197	Phenylalanine auxotroph λ^−^, e14-, pheA97, relA1, spoT1, thiE1	Hoekstra et al. [29]
*Escherichia coli* JW2580-1	Phenylalanine auxotroph *F* ^−^ *Δ(araD*-*araB)567*, *ΔlacZ4787*(::rrnB-3), *λ* ^−^, *ΔpheA762::kan*, *rph*-*1*, *Δ(rhaD*-*rhaB)568*, *hsdR514*	Baba et al. [30]
*Acetobacter nitrogenifigens* LMG 23498	Type strain. Cellulose producer	Dutta et al. [31]
*Acetobacter pasteurianus* Ap4	Strain recovered from healthy grapes microfermentation. Celullose producer	Valera et al. [32]
*Acetobacter syzygii* LMG 21419	Type strain. Cellulose producer	Lisdiyanti et al. [33]
*Acetobacter orientalis* LMG 21417	Type strain. Cellulose producer	Lisdiyanti et al. [33]
*Komagataeibacter europaeus* DSM 2004	Cellulose producer	Leibniz-Institut DSMZ, Germany
*Komagataeibacter hansenii* LMG 1524	Strain recovered from vinegar. Cellulose producer	BCCM LMG Collection, Belgium
*Komagataeibacter rhaeticus* LMG 22126	Type strain. Cellulose producer	Dellaglio et al. [34]
*Komagataeibacter europaeus* CECT 8454	Strain recovered from vinegar. Cellulose producer	This study

Vectors and constructs	Description	Reference or source
CopyControl pCC1FOS™	F-factor single-copy origin of replication and the inducible high-copy *ori*V	Epicentre Biotechnologies, Madison, WI
pBlueScript II SK (+)	Standard cloning vector (phagemid excised from lambda ZAP). The f1 (+) orientation allows rescue of sense strand ssDNA	Stratagene, La Jolla, CA, USA
pDrive	TA-cloning vector, *oriEc*, P_lac_ *lacZ*, Amp^R^, Kan^R^, T7-promotor	QIAGEN (Hilden, Germany)
pET21a	Expression vector, *lacI*, Amp^R^, T7-promotor, C-terminal His_6_-tag coding sequence	Novagen, Darmstadt, Germany
pET21a::*gqqA*	pET21a containing *gqqA* gene cloned into *Nde*I and *Xho*I restriction sites	This study
pBBR1MCS-5	Broad host range expression vector, *rep*, *mob*, *lacZ*, Gm^R^	Kovach et al. [35]
pBBR1MCS-5::*gqqA*	pBBR1MCS containing *gqqA* cloned into *Bam*HI and *Xho*I restriction sites	This study

Plasmid transformation in *E. coli* was carried out following a standard heat shock protocol [36] and by electroporation [37] in the case of *P. aeruginosa*.

The strain NTL4 of *Agrobacterium tumefaciens* [27], carrying a *traI*–*lacZ* promoter fusion in vector pCF372 [26] and extra copies of *traR* in vector pCF218 [25], was maintained at 30 °C in LB or AT medium [38] containing 0.5 % glucose per liter. Spectinomycin (final concentration 50 mg/ml) and tetracycline (final concentration 4.5 mg/ml) were added to maintain the vectors. The strain Cv026 of *Chromobacterium violaceum* was grown at 30 °C in LB medium. All AAB strains used in this study were grown in GY medium (1 % yeast extract, 5 % glucose) at 28 °C. When all these media were used as solid media, they were supplemented with 1.5 % agar.

### *Komagataeibacter europaeus* CECT 8546 strain fosmid library construction

The strain CECT 8546 of *Komagataeibacter europaeus* stored in our collection was recovered in GY medium (1 % yeast extract, 5 % glucose) at 28 °C for 48 h under shaking conditions (150 rpm). The genomic DNA of this strain was extracted with the DNA Isolation Kit for Cells and Tissues (Roche Diagnostics GmbH, Mannheim, Germany), and the Copy Control™ HTP Fosmid Library Production kit with the pCC1FOS™ Vector (Epicentre Biotechnologies, Madison, WI) was employed for its genomic fosmid library construction according to the manufacturer‘s instructions. The cells of the strain EPI300™ of *E. coli* were spread on LB agar medium with chloramphenicol and incubated overnight at 37 °C. The transformed colonies were transferred into 96-well microtiter plates containing 150 μl of LB medium with chloramphenicol and were incubated overnight at 37 °C. After this, 50 μl of 86 % glycerol was added to each well, and microtiter plates were stored at −70 °C.

### Screening for *N*-AHL-degrading clones using the NTL4 reporter strain of *Agrobacterium tumefaciens*

The fosmid clones from the genomic library of strain CECT 8546 were initially screened at least three times for their capacity to inactivate AHLs or to block AHL receptors/promoters. This AT soft agar screening was performed with the strain NTL4 of *A. tumefaciens*, which carries plasmid-based *traR* and a *traI*–*lacZ* promoter fusion. The activation of the *traI* gene is associated with the production of β-galactosidase (*lacZ* gene) which activity was detected using 5-bromo-4-chloro-3-indolyl-β-d-galactopyranoside (X-Gal) as the substrate [5, 39]. The concentration of 3-oxo-C8-HSL (Sigma-Aldrich, Heildelberg, Germany) used to supplement the soft AT soft agar medium was determined with a previous titration experiment without the presence of the fosmid clones. Concentrations from 10^6^ to 10^−4^ nM were tested, and 10 nM was determined to be the threshold concentration.

### Genetic analysis of positive clones and subcloning analysis

The genetic analysis of positive clones was carried out after AT soft agar screening to determine the correct transformation of fosmid clones with DNA from the strain CECT 8546. Ends of inserts, consisting of approximately 42 kb, were sequenced for fosmid clones that tested positive using PCC1-Fos Rev and T7 promoter primers (Table 2) and automated sequencing technology with the ABI3730XL DNA Analyzer (Applied Biosystems, Foster City, CA).

**Table 2:** Primers used in this study

Primer name	Sequence (5′ → 3′)	Reference
PCC1-Fos Rev	CTC GTA TGT TGT GTG GAA TTG TGA GC	Epicentre biotechnologies, Madison, WI
T7 Promotor	TAA TAC GAC TCA CTA TAG GG	Eurofins MWG Operon (Ebersberg, Germany)
M13-20 for	GTA AAA CGA CGG CCA GT	Eurofins MWG Operon (Ebersberg, Germany)
M13 rev	CAG GAA ACA GCT ATG ACC	Eurofins MWG Operon (Ebersberg, Germany)
gqqA Fw	CAT ATG AAC GGG GAA CGC ATC ATC	This study
gqqA Rv	CTC GAG GGG TTT GCG CCG GAA	This study

To detect the concrete open reading frames (ORFs) that are involved in QS inhibition, subcloning of fosmid clones was performed. The enzyme *Eco*RV (Fermentas, St-Leon-Rot, Germany) was used to obtain fragments that were ligated with T4 DNA ligase (Promega, Mannheim, Germany) in the plasmid pBlueScript II SK (+) and transformed into the *E. coli* strain DH5α.

All the clones produced by subcloning were again assayed for AT soft agar screening with the strain NTL4 of *A. tumefaciens* as described above. In addition, all of them were also tested for pyocyanin production assay with the strain PAO1 of *P. aeruginosa* as previously described by Gallagher et al. [40] and for a motility test with the transformed *E. coli* strain DH5α using swarming agar as described by Harshey and Matsuyama [41]. Cultures were grown for 16 h at 37 °C prior to the pyocyanin and motility assays; both analyses where carried out at least three times.

The positive clones in these screenings were then completely sequenced using primers M13-20 for and M13 rev (Table 2). Gaps were closed by primer walking. Nucleotide and amino acid sequence comparisons were carried out using the BLAST program [42] and the GenBank database.

### Purification of His-tagged proteins

Considering the results obtained from the screening with reporter strains and sequencing, an ORF named *gqqA* was selected. It was amplified using the primer pairs gqqA Fw and gqqA Rv (Table 2), and the fragment was initially cloned into the pDrive vector and then was excised and cloned into the expression vector pET-21a. Both amplicon and vector were digested with *Nde*I and *Xho*I and ligated directionally, yielding pET21a::*gqqA*. This construct was transformed into the strain BL21 (DE3) of *E. coli*, which was grown at 37 °C in LB medium with ampicillin to an OD600 of 0.5–0.8. Expression was induced by the addition of 0.8 mM IPTG (isopropyl-β-d-1-thiogalactopyranoside) (Sigma-Aldrich, Heildelberg, Germany), and cultures were incubated overnight at 28 °C and 150 rpm. Cells were harvested by centrifugation at 10,000 rpm for 15 min and 4 °C and resuspended in LEW buffer (50 mM NaH_2_PO_4_; 300 mM NaCl). Cell disruption through a French press was performed three times at 1100 Bar, and the lysate was centrifuged at 15,000 rpm for 15 min and 4 °C. The supernatant obtained was purified using Protino Ni-TED 2000 packed columns (Macherey–Nagel, Dueren, Germany) following the manufacturer’s protocol.

Moreover, the lactonase QsdR1 from the strain NGR234 of *Rhizobium* sp. was used as a positive QQ control for its activity as QS signal degrading enzyme. This protein was purified from the overproducer strain BL21 (DE3) of *E. coli* containing the vector pET21a::*qsdR1* as described by Krysciak et al. [19].

In both cases, the protein purity and molecular mass were determined by SDS-gel electrophoresis and the concentration using the Bradford protein assay [43].

### Effect of the GqqA protein on β-galactosidase activity using the reporter strain NTL4 of *Agrobacterium tumefaciens*

The ortho-nitrophenyl-β-d-galactopyranoside (ONPG) test was also carried out using the *A. tumefaciens* strain NTL4 and the purified GqqA protein extracts. Both *N*-3-oxooctanoyl-l-homoserine lactone (3-oxo-C8-HSL) and *N*-3-oxododecanoyl-l-homoserine lactone (3-oxo-C12-HSL) (Sigma-Aldrich, Heildelberg, Germany) were tested in triplicate as previously described [39] with minor modifications. Briefly, 5 μl of 10 nM and 100 nM AHLs were added to 100 μl of purified GqqA protein extract (1 mg/ml) and incubated at 30 °C in 100 mM potassium phosphate buffer at pH 8.0. After incubation with the strain NTL4 in AT medium, 1 ml of cell suspension was mixed with 20 μl of toluene and vortexed for 3 min. This solution (800 μl) was mixed with 200 μl of the ONPG solution and incubated for 20 min at room temperature before measuring the absorbance at 420 nm.

Negative controls were performed using bovine serum albumin (BSA) (Sigma-Aldrich, Heildelberg, Germany) and protein extract from *E. coli* obtained as described above for the purification of GqqA but using the strain *E. coli* BL21 (DE3) with the plasmid pET-21a recircularized, instead of the GqqA protein at the same final concentration.

### Effects of the GqqA protein on swarming motility and pyocyanin production of the reporter strain PAO1 of *Pseudomonas aeruginosa*

To analyze the effects of the *gqqA* gene on the motility and pyocyanin production of the *P. aeruginosa* strain PAO1, this gene was cloned into the broad host range vector pBBR1MCS-5. The ORF was amplified using the primer combination gqq Fw and gqq Rv (Table 2), and the pBBR1MCS-5::*gqqA* construct was transferred into the strain PAO1 by electroporation.

The swarming motility test was performed in agar with M9 medium [36] and 0.05 % glutamic acid but without NH_4_Cl and solidified with 0.5 % Eiken Agar (Eiken Chemical, Tokyo). One microliter with 1 × 10^7^ cells of an overnight PAO1 strain culture was applied to the middle of the agar plate. The swarming phenotype was documented by photography after incubation at 37 °C for 16 h. The pyocyanin production was measured using the protocol reported by Gallagher et al. [40]. Analyses performed in triplicate were compared with controls using the strain PAO1 carrying the recircularized vector pBBR1MCS-5.

### Effects of the GqqA protein on violacein production by the reporter strain Cv026 of *Chromobacterium violaceum*

The strain Cv026 of *C. violaceum* was also used to analyze the effects of purified GqqA protein. A volume of 15 μl of purified GqqA protein at 2 mg/ml was incubated separately with three different concentrations of C6-HSL (Sigma-Aldrich, Heildelberg, Germany), 10^3^, 10^2^ and 10 μM. After incubation for 3 h at 30 °C, this mixture was added to a tube containing 2 ml of LB medium and 10 μl of the strain Cv026 culture and was incubated for 20 h at 30 °C and 150 rpm.

Two different controls were used: in one of them, the molecule of C6-HSL was not added to determine the residuary production of violacein by the Cv026 strain, and in the other, GqqA protein was substituted by BSA at the same concentration. Violacein production in triplicate was performed and photographed; the absence or impairment of purple coloration indicated a lack of QS activity.

To quantify the amount of violacein produced with each treatment, the protocol reported by Hornung et al. [44] with minor modifications was used. Two milliliters of grown culture were centrifuged for 2 min at 12,000*g* and concentrated in 0.4 ml of water. One volume of sodium dodecyl sulfate was added, and after being incubated at room temperature for 5 min, the lysate was precipitated with 0.9 ml of 100 % ethanol. This extract was centrifuged at 13,000*g* for 5 min. The absorbance of the supernatant was determined at 575 nm, and the amount of violacein formed was expressed in relation to the OD600 that was measured for each sample.

### Effects of GqqA protein on growth and the cellulose production phenotype of AAB

The effects of the GqqA protein were also tested both with the strain CECT 8546 of *Komagataeibacter europaeus* and in seven other cellulose-producing strains of AAB, four belonging to the *Acetobacter* and three to the *Komagataeibacter* genera (Table 1). A culture of each strain was obtained in GY medium following incubation for 48 h at 28 °C.

For analysis of the CECT 8546 strain, 50 μl of its culture was inoculated in tubes with 5 ml of GY medium and with three different concentrations of the purified GqqA protein extract (5, 10, and 20 μg/ml). Controls were performed with BSA and protein extract from *E. coli* at three concentrations (5, 10, and 20 μg/ml). All conditions were performed in triplicate. The growth of strain CECT 8546 was monitored at 16, 24, 48 and 72 h by measuring the OD600 of the medium, microscope counting, plating onto GY solid medium and visual inspection. Moreover, at these sampling points, the sugar consumption of the strain for these two conditions (BSA and GqqA added) was measured using an enzymatic kit for d-glucose quantification (Boehringer, Mannheim, Germany) following the specifications of the manufacturer. In addition, epifluorescence microscopy was used to compare the evolution in the growth of this strain after 16 h. A volume of 10 μl from each sample was stained with 1 μl of SYTO9 dye and 1 μl of propidium iodide (PI) dye from the Live/Dead BacLight Kit (Molecular Probes, Eugene, OR, USA). After incubation in the dark for 20 min, each sample was washed with 2 μl of water to eliminate the excess dye and observed under epifluorescence microscopy.

For the analysis of the cellulose production phenotypes of other AAB strains, 50 μl of each strain culture was inoculated into 24-well microplates with 2 ml of GY medium supplemented with 100 μg of the purified GqqA protein extract (50 μg/ml). Controls were carried out with 100 μg of BSA and with 10 μg of protein extract from *E. coli* instead GqqA protein. All the conditions were analyzed in triplicate. The results were photographed after 16 h.

The growth phenotype observed in the presence of the GqqA protein was also analyzed in the presence of the lactonase QsdR1, which was previously characterized as having strong QQ activity [19]. To carry out this test, 100 μg of purified QsdR1 protein (20 μg/ml) was added to 5 ml of GY medium previously inoculated with 50 μl of an overnight culture. This analysis was carried out for the strains CECT 8546 and *Komagataeibacter rhaeticus* LMG 22126.

Moreover, to test if GqqA protein exerts an effect on the AHLs and is involved in the mechanism for growth and the cellulose production phenotype of the strains CECT 8546 and *Komagataeibacter rhaeticus* LMG 22126, a mixture of three AHL molecules at high concentrations was added. The strains were inoculated in 5 ml of GY medium and 100 μg of the purified GqqA protein (20 μg/ml) in addition to the AHL molecules *N*-oxo-C8-l-HSL, *N*-C10-l-HSL and *N*-C12-l-HSL (Sigma-Aldrich, Heildelberg, Germany) with a final concentration of 50 μM for each. Moreover, for strain CECT 8546, the effects of these AHL molecules were also studied individually using final concentrations of 5 and 10 μM for each. All the conditions were analyzed in triplicate. The results were photographed after 24 h.

### Complementation assays with auxotrophic strains of *Escherichia coli* and auxotrophy analysis of the *Komagataeibacter europaeus* CECT 8546 strain

A complementation test was carried out with two auxotrophic strains of *E. coli* for phenylalanine, which were obtained from the Coli Genetic Stock Center (http://www.cgsc.biology.yale.edu). Strain JW2580-1, which contains an in-frame, single-gene knockout from the Keio Collection [30], and the strain KA197 [29] are described in Table 1. These strains were recovered in LB medium overnight at 37 °C. Their phenylalanine auxotrophy was checked by growing on the minimal medium M9 supplemented with and without d-phenylalanine (10 μM) (Sigma-Aldrich, Heildelberg, Germany).

Both strains were transformed with the constructed vector pET21a::*gqqA* following the protocol described by Dagert and Erlich [45]. These transformed strains as well as auxotrophic strains were grown in LB medium overnight at 37 °C and at 150 rpm; then, 5 ml of each culture was centrifuged, and cells were washed with sterile water and recovered after centrifugation in 1 ml of sterile water. Then, 75 μl of a 1:100 dilution was plated onto M9 medium with and without phenylalanine.

On the other hand, an auxotrophy assay for phenylalanine with the strain *Komagataeibacter europaeus* CECT 8546 was performed using 1.7 g/l of a minimal medium based on Yeast Nitrogen Base without amino acids and ammonium sulfate (Difco, Detroit, MI) supplemented with 50 g/l of d-glucose and 1 g/l of ammonium sulfate. Moreover, all the amino acids except for phenylalanine were added into the minimal medium as reported by Ameyama and Kondo [46] for the requirements of AAB. A control was performed by also adding phenylalanine to the medium to favor the growth of the strain.

### Accession numbers

The nucleotide sequence of the *gqqA* gene was deposited in the GenBank, National Center for Biotechnology Information, http://www.ncbi.nlm.nih.gov/nucleotide. It corresponds to locus tag KOEU_05990 of *Komagataeibacter europaeus* strain CECT 8546 KOEU_contig000002, whole genome shotgun sequence, NCBI Reference Sequence: NZ_LHUQ01000002.

## Additional file

10.1186/s12934-016-0482-y A) Restriction patterns of 10 fosmid clones from the genomic library of the strain CECT 8546 of *Komagataeibacter europaeus* obtained with *Bam*HI enzyme (lanes 1–10); GeneRuler 1 Kb DNA ladder (Thermo Scientific) (lane M). The probability of containing all the genome within the whole library was 99.99 %, as calculated according to the manufacturer’s instructions. B) SDS-PAGE image of the GqqA protein. Lane M: molecular marker [unstained protein ladder (Fermentas)]; Lane 1: purified GqqA his-tagged protein (5 μg). Lane 2: protein extract from transformed strain BL21 (DE3) of *E. coli* induced for GqqA overexpression. C) Map of the predicted ORFs in the fosmid clone selected for QQ activity, which contained the *gqqA* gene. The position and the direction of transcription are indicated for ORFs.
