# Supplementary material for: GqqA, a novel protein in Komagataeibacter europaeus involved in bacterial quorum quenching and cellulose formation
Source: Microb Cell Fact. 2016 May 24;15:88. doi: 10.1186/s12934-016-0482-y (PMC4879726; doi:10.1186/s12934-016-0482-y)
Supplement: Supplementary file 1 — 10.1186/s12934-016-0482-y A) Restriction patterns of 10 fosmid clones from the genomic library of the strain CECT 8546 of Komagataeibacter europaeus obtained with BamHI enzyme (lanes 1–10); GeneRuler 1 Kb DNA ladder (Thermo Scientific) (lane M). The probability of containing all the genome within the whole library was 99.99 %, as calculated according to the manufacturer’s instructions. B) SDS-PAGE image of the GqqA protein. Lane M: molecular marker [unstained protein ladder (Fermentas)]; Lane 1: purified GqqA his-tagged protein (5 μg). Lane 2: protein extract from transformed strain BL21 (DE3) of E. coli induced for GqqA overexpression. C) Map of the predicted ORFs in the fosmid clone selected for QQ activity, which contained the gqqA gene. The position and the direction of transcription are indicated for ORFs. [file 12934_2016_482_MOESM1_ESM.ppt]

## Slide 1
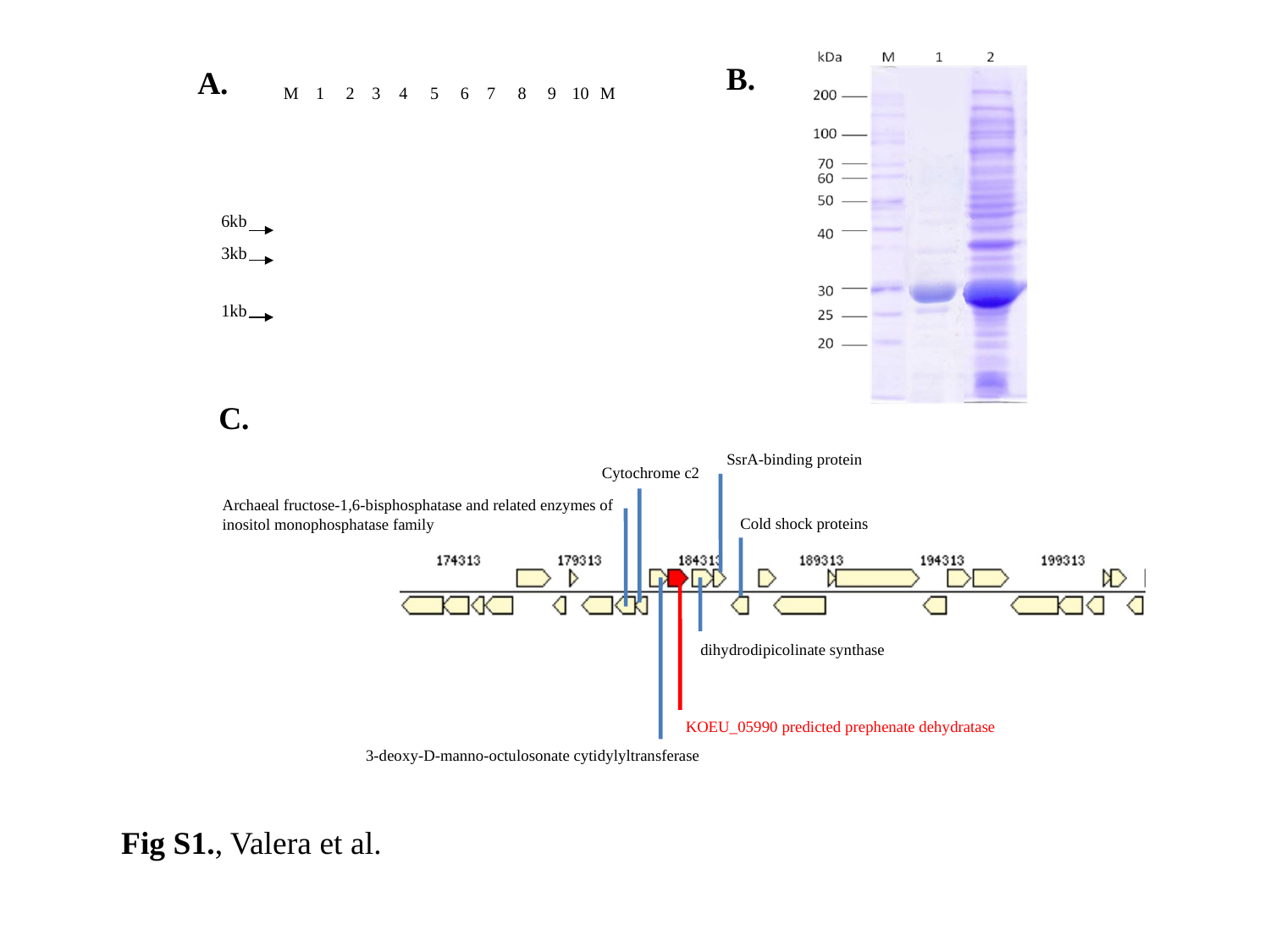

B.
A.
C.
SsrA-binding protein
Cytochrome c2
Archaeal fructose-1,6-bisphosphatase and related enzymes of inositol monophosphatase family
Cold shock proteins
dihydrodipicolinate synthase
KOEU_05990 predicted prephenate dehydratase
3-deoxy-D-manno-octulosonate cytidylyltransferase
Fig S1., Valera et al.
